# Social Media Engagement in Two Governmental Schemes during the COVID-19 Pandemic in Macao

**DOI:** 10.3390/ijerph19158976

**Published:** 2022-07-23

**Authors:** Patrick Cheong-Iao Pang, Wenjing Jiang, Guanwen Pu, Kin-Sun Chan, Ying Lau

**Affiliations:** 1 Faculty of Applied Sciences, Macao Polytechnic University, Macao, China; mail@patrickpang.net; 2 Center for Social Security Studies, Wuhan University, Wuhan 430072, China; wenjingjiang@whu.edu.cn; 3 Faculty of Business Administration, University of Macau, Macao, China; mc04665@um.edu.mo; 4 Faculty of Social Sciences, University of Macau, Macao, China; 5 Grand Thought Think Tank, Macao, China; 6 Alice Lee Centre for Nursing Studies, Yong Loo Lin School of Medicine, National University of Singapore, Singapore 117597, Singapore; nurly@nus.edu.sg

**Keywords:** content analysis, COVID-19, Facebook, governmental scheme, social media

## Abstract

Social media engagement is a vehicle for effective communication and engagement between governments and individuals, especially in crises such as the COVID-19 pandemic. Additionally, it can be used to communicate resilience measures and receive feedback. This research aims to investigate public social media engagement with resilience measures related to COVID-19 in Macao. We examined 1107 posts and 791 comments about the government’s face mask supply and consumption voucher schemes on Facebook. Using the Crisis Lifecycle model, we partitioned the data and analyzed the content and engagement of related posts, as well as the word semantics in user comments. Our findings show that social media engagement in these resilience measures is high and positive in the early stages of the pandemic, suggesting social media’s potential in mobilizing society, preserving social resilience, and serving as a two-way communication tool in public health emergencies.

## 1. Introduction

The ongoing global COVID-19 pandemic has brought substantial numbers of infections and deaths worldwide and at the same time affects the global economy [1]. With the rapidly changing and uncertain nature of the SARS-CoV-2 virus, it is important for governments and public health authorities to react promptly and share accurate information with the public [2] to let individuals participate in fighting against the virus. Among the different platforms, social media is widely used to facilitate communication between governments and individuals [3]. An increasing number of studies have investigated the effect of social media on the prevention of COVID-19 and raising awareness of the public [4,5], vaccinations [6,7], and dispelling COVID-19 controversies [8,9,10].

COVID-19 affects public health, disrupts the supply chains of daily and medical supplies, and affects local economies, hence preserving social resilience for future recovery is an important post-COVID-era task [11]. Here, we define the concept of resilience as the “ability to recover from a crisis and bounce back” as defined in [12], and the literature shows that governments have taken different resilience measures with the hope of returning to normal [13,14,15]. Although researchers have, on one hand, been focusing on the governments’ use of social media for public health measures, citizen engagement on social media on resilience and financial support measures on the other hand is under-researched. It is also unclear whether social media is useful when communicating governments’ responses on socio-economic recovery. As such, this research aims to look at public Facebook engagement toward resilience measures during the pandemic, using the COVID-19 responses from the two government schemes of the Macao Special Administrative Region (SAR) Government.

Our interests focused on the face mask supply scheme [16] and the consumption voucher scheme [17] that were provided by the Macao Government during the COVID-19 pandemic. These schemes were selected because of their substantial and controversial nature during the COVID-19 pandemic [18]. Face masks, as basic protection, were perceived to reduce the risk of infection at the beginning of the pandemic [19]. Due to the preliminary lack of supplies, it was difficult to obtain face masks amid hoarding and speculation [20]. Meanwhile, the Macanese tourism, hotel, and gaming industries were severely affected, and enterprises were forced to a halt [21]. The subsequent restrictions further caused the paralysis of the economy. Hence, consumption vouchers were considered as the government’s attempt to revive the economy by addressing needs on living costs and increasing residents’ purchasing power [16,17]. Both schemes are considered as public health emergencies for all Macanese residents.

This paper leverages the different stages in Fink’s Crisis Lifecycle model [22] to examine the use of social media by the Macao Government at different time points in a public health emergency, as well as how the public responded and engaged its resilience measures. The Crisis Lifecycle model defines four stages: prodromal, acute, chronic, and resolution. The prodromal stage develops before the occurrence of crises, where the focuses include the management of potential hazards and vulnerabilities and strengthening guidelines for the rectification of hidden dangers. In the acute stage, crises grow rapidly, and the main goals are to monitor, track, and control the crisis, with the hope to avoid its deterioration. The chronic stage signals the ease of crises which requires reducing damage, effective coordination, and maintaining resources (such as budget and manpower) to keep controlling the situations. Finally, the crises are concluded in the resolution stage where governments should investigate and learn lessons from the aftermath and avoid future crises. Each stage in this model has its purposes, and therefore our motivation is to investigate the different use and public engagement of social media at these stages.

Facebook, as a popular social media platform, is an important venue for communication between the government and the larger public [23]. A local survey found that nearly 70% of Macao Internet users use Facebook [24], and the Macao Government has been using Facebook as one of the channels to share information. Therefore, it can be seen as a probe for analyzing the reception of resilience measures in Macao during public health emergencies [25]. Informing citizens of critical information on social media relieves the concerns of local communities thus increasing social resilience [11]. Social media has played a role in communication and mobilization [4] as an effective two-way communication tool during public health crises [26]. A better understanding of how social media engagement is formed will be beneficial to handling future public health crises using social media.

## 2. Background

Macao, a populated city in Southern China, has a micro-economy and is renowned for its gaming and tourism industry [27]. This economic structure makes it prone to the impacts brought by regional and global public health measures against COVID-19 [28]. Measures such as city lockdowns and travel restrictions have significantly reduced the number of tourists [12,29]. This heavy blow to the hospitality industry was reported shortly after the emergence of COVID-19 in nearby regions [14,29], and a small city like Macao with a high population density could lead to a high number of infections [21]. To avoid this, the Macanese Government took stringent approaches to prevent and control the virus in its early stages [30]: A 24/7 COVID-19 Coordination Center was established with the Chief Executive of the Macao SAR as the chairman [15,23], and border control was imposed in the first half of 2020, which resulted in the city’s COVID-free status for 130 consecutive days [12].

When the COVID-19 pandemic reached the city, previous experience suggested that using a face mask reduces the likelihood of contracting the virus [31] and was therefore enforced in public spaces such as public transportation, schools, and government offices. However, the supply of face masks was constrained in the early parts of 2020 [21]. The outbreak in China also greatly reduced the efficacy of face masks, with the consequent rise in infection numbers causing panic buying among residents [20]. Here, the government introduced a face mask supply scheme [16] to integrate its procurement, quality control, and sales. As a result, residents could purchase face masks with guaranteed availability and prices, effectively preventing looting and market speculation [20]. This measure is one of our research’s focuses given its perception as a critical response by the government at the earlier stage of the epidemic to mobilize and stabilize individuals [20,21].

Strict border restrictions and other preventive measures undoubtedly affected the Macanese residents and economy [21]. The gross revenue from gaming, one of its major industries, dropped substantially by about 80% in 2020 with the pandemic [32], and the occupancy rate of hotels fell to approximately 15% [29]. Ultimately, the Gross Domestic Product (GDP) of Macao dropped by nearly 50% in the first quarter of 2020 [29]. To stir socio-economic resilience and recovery, the government launched multiple rounds of consumption voucher schemes [17], with the first round launched in mid-2020. The initial consumption voucher scheme offered eligible residents an electronic cash voucher with MOP 3000 (equivalent to USD 375) to spend locally for three months, injecting a total consumption power of MOP 2.2 billion (USD 275 million) into the local market [33]. The scheme was successful in reviving the consumer market and business environment and was considered a key policy in maintaining social resilience and the recovery of both the tourism sector and the local economy [34]. We studied the engagement of this measure given its pivotal role in supporting the economic needs of the community, which can illustrate the engagement on social media from an angle different from public health in the middle of the outbreak.

## 3. Methods

User-generated online data such as social media and online health community posts can reflect the views, preferences, and behaviors of people [35,36]. Given the increased usage of social media during the pandemic [37] and the popularity of Facebook in Macao [24], this work focuses on the social media engagement on this platform. Below explains how we collect Facebook data, partition data according to crisis stages, and perform different analyses on the data.

### 3.1. Defining Crisis Stages

Based on the Crisis Lifecycle model [22], the entirety of 2020 was divided into stages and analyzed accordingly. The divisions used were the prodromal, acute, and chronic stages. The resolution stage is not used in this work as the global pandemic continues and has not reached its end. The definitions of the crisis stages used in this paper are listed in Table 1. For the acute stage in this event, the end of an outbreak (i.e., the end of the stage) can only be declared when there are no further infections after two incubation periods (28 days of the original strain of SARS-CoV-2) [23,38].

### 3.2. Data Collection

The researchers used a customized Python script to crawl posts and comments with the keywords related to face masks and consumption vouchers from Facebook every day. The range of data collection included the Government’s official Facebook pages and the pages of large local news outlets. The duration of data collection was from 1 January 2020 to 31 December 2020. 1107 posts and 791 comments relating to the abovementioned two schemes were collected.

### 3.3. Data Analysis

In this study, three data analysis methods were used, including content analysis, social media engagement analysis, and word frequency & semantic network analysis. We performed content analysis on posts and engagement analysis by totaling the likes, shares, comments, and other reactions [6,39]. The content analysis approach studied complex public health phenomena by transforming a large amount of textual data into systematic themes and categories [39], which is useful for us to extract key themes from the posts and understand what topics were covered.

Next, we evaluated the engagement of the posts that were relevant to the policies to gauge the posts’ efficacy in public communication. Consistently [23,26], the engagement on social media was measured by the sum of the four different emotions expressed by Facebook users, with details listed as follows:

(1) Positive emotions which included likes, love, and care emotions expressed along with the posts
(2) Negative emotions which included angry and sad emotions
(3) Numbers of comments which reflected the commitments and motivations of interactions and discussions
(4) Numbers of shares, which reflected content recognition and reach.

Since the comments on the Facebook posts could reflect their areas of concern and their thoughts on the measures, we performed word frequency analysis to highlight the outstanding keywords among these comments [40]. To further examine the relationships among words and understand their substantive meanings, we also conducted a semantic network analysis of words [41]. We employed popular word segmentation libraries in Python: PKUSEG [42], NLTK toolkit [43], and NetworkX [44] to calculate word occurrence and perform the creation of word semantic networks. Inspired by [45], the top 10 words with the highest frequencies were used. Eigenvectors of words were calculated to denote the importance of words in the dataset.

## 4. Results

This section presents the results of our analysis. Our analysis is based on Chinese because it is the language used by most Macanese. For the purpose of reporting, the content presented below was translated into English by the authors.

### 4.1. Content Analysis of Posts

Our content analysis revealed six main themes. It was reported that the economic measures could support struggling citizens and businesses, especially small and medium-sized enterprises. Another reporting theme showed government efforts to source and secure protective gear (such as face masks) for citizens and distribute it to those in need. In addition, as part of the policy implementation, the government refined and adjusted its policies through inter-departmental cooperation. Citizens mostly agreed and felt grateful for these responses. Table 2 displays the list of the themes identified from the data.

### 4.2. Engagement Analysis of Posts

The results demonstrate a micro perspective of the receptions of the abovementioned government responses in the prodromal, acute, and chronic-resolution stages. The diagram displayed the average values of the four metrics (i.e., positive, negative, comments, and sharing counts) in log scale along with the key development of the COVID-19 outbreaks in Macao.

#### 4.2.1. Engagement of the Face Mask Supply Scheme

**Figure 1:**
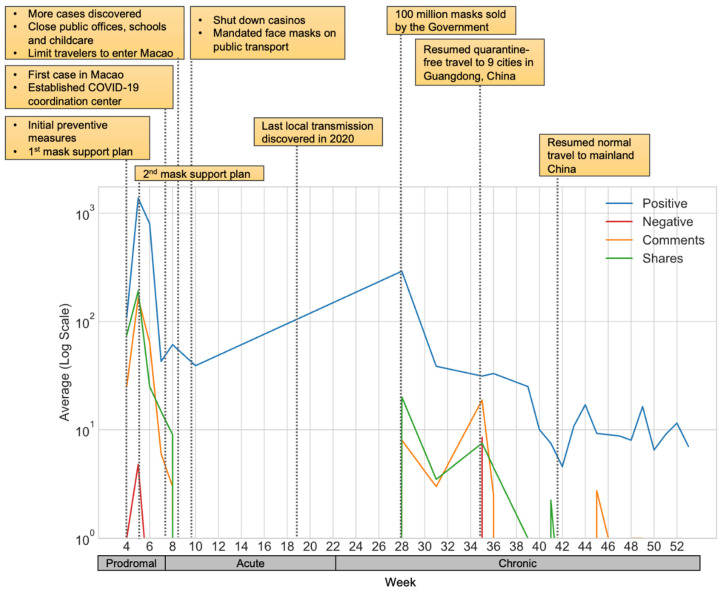
The engagement of the face mask supply program in the epidemic (*X*-axis denotes calendar weeks in year 2020; *Y*-axis is displayed in log scale; shaded boxes list key events in the epidemic).

Figure 1 presents the engagement related to face mask supply along with the key events in the epidemic. As shown in the prodromal stage when the first and the second rounds of the face mask supply scheme were announced, the public demonstrated strong engagements with the government with a sharp increase. Among these, the highest metric was positive emotion, followed by the number of shares, and then the number of comments. These metrics showed a clear difference from negative emotion, observed as low on face mask-related content. In the late prodromal and the early acute stages, however, the trend turned downwards while the government set up the coordination center and the first patient was subsequently identified.

As the acute stage revealed more COVID-19 cases, the government enforced various measures such as closing public venues, workplaces, schools, border controls for non-residents, and even the suspension of casinos which are the economic lifeblood of the city. Here, the engagement with face mask measures decreased, particularly for sharing and commenting. After the last local case in 2020 was recovered, positive engagement about face masks started to consistently increase in the late acute stage. The engagement also increased when the face mask scheme reached a milestone when 100 million masks were sold. Moreover, in the chronic stage, positive engagement could be seen as one apparent reaction, and some negative engagement with higher numbers of shares and comments appeared after the resumption of tourist entries from nine cities in the province of Guangdong.

Ultimately, face mask-related positive engagements were far higher than negative ones during the pandemic, and the numbers of shares and comments showed greater values in both the acute and chronic stages. The engagement on face mask measures suggested that the public was generally satisfied with the responses from the Macao SAR government. Each round of the face mask supply scheme was actively disseminated to the public despite the initial phase of the program being limited by experience and supply chain problems, which caused a relatively small number of negative reactions.

#### 4.2.2. Engagement of the Consumption Voucher Scheme

**Figure 2:**
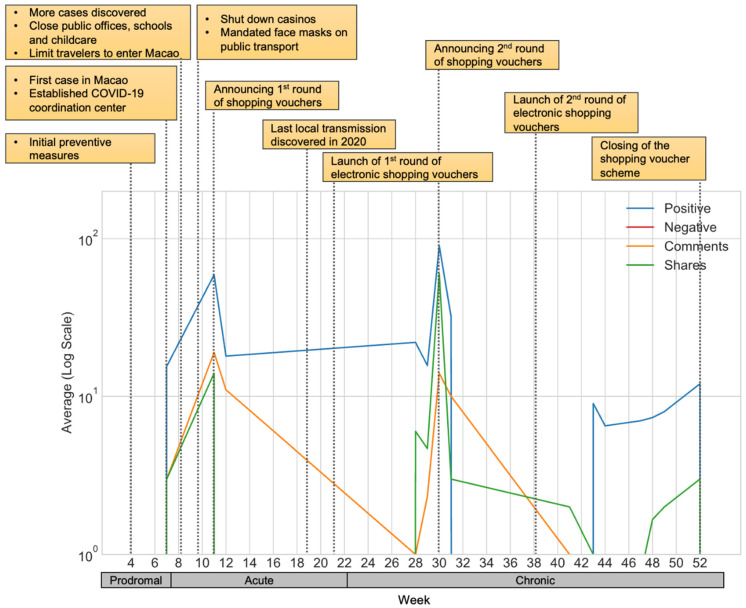
The engagement of the consumption voucher program in the epidemic (*X*-axis denotes calendar weeks in year 2020; *Y*-axis is displayed in log scale; shaded boxes list key events in the epidemic).

Figure 2 displays the trend of engagement on consumption vouchers with key events listed in the figure. The social media engagement on consumption vouchers appeared later than the face mask scheme because the former was announced after. From the first COVID-19 case until the scheme’s announcement, public participation was reflected by an upward trend, especially for positive emotion, because of the residents’ anticipation of economic support. The numbers of shares and comments were found to also be similar. From the mid-late acute to the early chronic stage, when zero local acquired cases were achieved and the consumption voucher scheme started, the positive engagement of the scheme was observed to be constant. Despite this, the number of comments decreased until it reached almost zero.

In the middle of the chronic stage, the announcement of the second round of consumption vouchers caused a high number of positive engagement and shares, accompanied by a high number of comments. The trend remained upward until the end phase of this stage when the consumption voucher scheme was closer to its termination. Essentially, residents’ participation during the entire scheme was positive. The sharing and commenting trends mirrored the trend of positive reaction, while negative emotions could hardly be seen. The low negativity, high participation, and proactive sharing of the information reflected the wide acceptance of consumption vouchers and showed the great level of support and recognition of Macao’s public health emergency responses.

### 4.3. Word and Network Analysis of Comments

**Figure 3:**
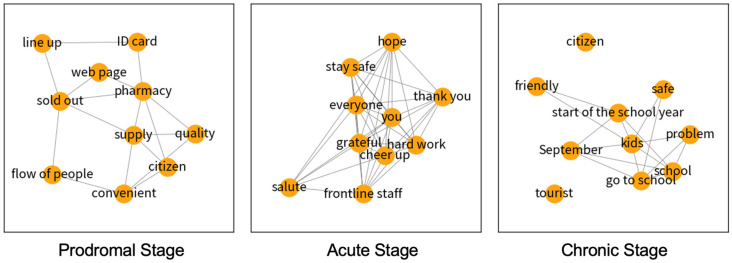
Network diagrams of the face mask scheme in three stages.

Table 3 presents the 10 most recurring words in user comments regarding the face mask supply program during different stages, accompanied by their eigenvector values. In the prodromal stage, the comments generally focused on the mask and its supply (such as where to purchase, convenience, and the amount of supply). During the crisis, masks became a public product that needed to be state-organized. Therefore, Macanese citizens were anxious about the sale of protective face masks. In addition, the maintenance of social order (for example, lining up and the use of ID cards when purchasing), as well as the quality of masks, received extensive public scrutiny. In the acute stage, the comments shifted to care and appreciation for frontliners, which showed local support for the policies. In the chronic stage, the discussion centered on arrangements for the resumption of classes and childcare, adjacent to the safety of children resuming school. These were reflected by the word network diagram (Figure 3) as words such as “kids”, “safe” and “go to school” were connected.

**Figure 4:**
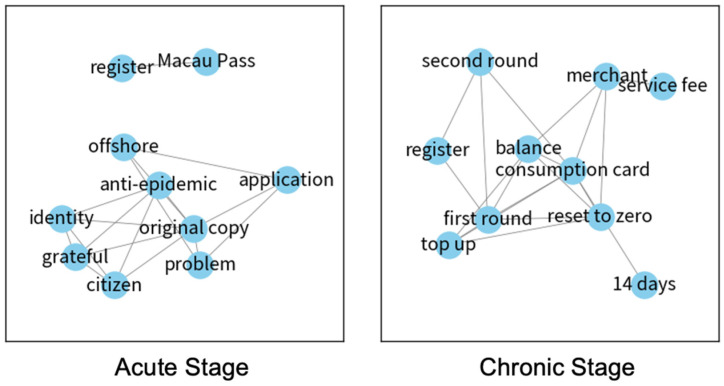
Network diagrams of the consumption voucher scheme in the acute and chronic stages.

Table 4 presents the 10 most recurring words in user comments on the consumption voucher scheme. Due to the policy’s late introduction, related comments only started to appear in the acute stage. Here, the public focused on program registration and the use of consumption vouchers (in the form of Macau Pass, which was an electronic payment card). Additionally, the discussion involved issues such as the coverage of the vouchers, eligibility, and the procedure of registration. Followed by the chronic stage, citizens shifted toward the issue of multiple rounds of consumption voucher programs, such as topping up electronic voucher cards for reuse and merchant service fees involved in transactions. Figure 4 displays the relationships of words used in user comments, which clearly illustrates the relevance of “merchant” and “service fee”, and how to “register” for the “first round” and the “second round” of the scheme.

## 5. Discussion

Our analysis revealed that the two supporting measures after the emergence of COVID-19 gained a high level of engagement on social media in different stages of the 2020 outbreak in Macao. Among these engagements, the number of comments and shares also reached a high level after the announcement of two supportive measures. This suggests that the public both read pandemic-related information and made use of said information with commitment, interaction, and reach. Hence, we argue that social media can act as an effective and engaging channel to communicate resilience measures to the public during public health emergencies. This bears significance given how governments could easily lose public trust in extraordinary times, which may lead to a lack of community participation in disaster relief work [46].

Further, with the evidence of post-traumatic stress disorder from time to time in recent years, social solidarity has become essential in coping with post-disaster reconstruction to build community resilience in emergency management [47]. With public and private entities having access to social media, the platform can enable effective mobilization and information communication during pandemics. With Macao as an example, communities and local groups play a vital role in mobilization. These organizations provide services targeted at different social sectors. Using the “government-owned, privately operated” principle, the role of traditional associations in Macao in the development of social services cannot be overstated [48]. Echoing emergency projects led by the Latino immigrant community in the United States, traditional associations can play a similar role in building community resilience and social cohesion to speed up communal recovery after a disaster [49]. Our study shows that these associations can increase their use of social media during public health emergencies to preserve social resilience and convey protective and supportive health-related messages.

Government-issued information is sparse during times of crisis. When responding to crises such as pandemics, governments need to clarify misinformation and let the public understand the current situation. Therefore, it is crucial for trustworthy information to be released by officials and consolidate the use of social media through government and community-led projects, which can be facilitated by the Macanese government. Moreover, dispelling hearsay is another concern of emergency management, which is why agencies such as the United States Federal Emergency Management Agency introduced Coronavirus Rumor Control to help the public distinguish between rumor and fact [50]. Other studies also advocate the importance of rumor control and regulation on social media during emergency periods [23].

As shown by the keywords of user comments on Facebook, the Macao Government has gained the recognition of the public regarding the supply of masks in the COVID-19 response. The assurance of material supply for the public has relieved the panic in society caused by the burst of rigid demand in the pandemic. Making appropriate arrangements while prompting citizens to expect an ongoing trend of the pandemic by having multiple stages of supporting programs is one of the reasons why the government enjoys great engagement over time. A dedicated and well-established social media page can act as a long-term communication tool with the public for pandemic information and resilience measures. This knowledge should be applicable to other countries/regions not only for public health crises but also for other types of emergency management.

Apart from appreciation comments, keywords related to daily lives and the implementation of policies (e.g., back-to-school arrangements for children) can also be seen at different stages in our analyses. This reflects that the public actually used social media as a channel to provide feedback and suggestions to the government as the pandemic evolved. Research has found that governments tend to only carry out one-way communication (such as announcements) on social media [51], and such an approach is inappropriate and does not meet citizen expectations in times of crisis. During unprecedented times, public health responses need to adjust rapidly, and social media can serve as a probe to understand how society reacts to these responses. Social media as a two-way communication channel between governments and the public will continue to play a crucial role in public health emergencies.

## 6. Limitations

Our approaches used in this study have two main limitations: our work is based on a single case study on Macao and therefore may not be directly applicable to other places due to different structures of governance. Also, we only collected data from a single social media application, which is Facebook. While it is the most popular social media platform in Macao, opinions on other platforms may be unrepresented. However, these may show the future extension of our research to examine other systems of governance and other governments worldwide using data from multiple social media websites.

## 7. Conclusions

As the threats of public health emergencies arise, it is critical to have solid communication to control the spread of crises and protect lives. With thorough analysis using Facebook data, we have reviewed the public engagement on social media after the Macao SAR Government announced and implemented policies of assisting with social resilience. Our analysis suggests that social media can provide considerable levels of engagement for COVID-19-related responses at different stages and can be effective for two-way communication during public health crises with fast-changing situations.

## Figures and Tables

**Table 1:** Crisis stages of the first COVID-19 outbreak in Macao.

Crisis Stage	Definition	Start Time	End Time
Prodromal	This comes before the actual crisis, and its focus is to prevent or delay the crisis from happening.	The start of the current analysis	The first confirmed COVID-19 case
Acute	After the prodromal stage, the acute stage is signaled by the sudden onset of the event, and the event often develops rapidly. The main goals lie in controlling the crisis and avoiding its deterioration.	The first confirmed case	28 days after the last case of local transmissions
Chronic	The crisis situation begins to ease in this stage and its appearance is less dramatic in appearance. As such, the focus should be on relieving controlling measures, reducing damage, and initiating the steps towards recovery.	The first day after the acute period	The end of the current analysis
Resolution	The crisis is over at this stage. Learnings should be synthesized for preparing the responses to future crises and the society/organization is returning to normal.	Not applicable (since the world is still in the middle of the pandemic at the time of writing, this stage is not applicable).	

**Table 2:** Themes identified from content analysis.

Theme	Sample Quotation
Supporting small and medium enterprises	“It is hoped to attract consumers to small and medium-sized enterprises by issuing electronic consumer vouchers with targeted functions to maintain consumption momentum and expand domestic demand … and support the development of small and medium-sized enterprises and merchants”
Supporting families and citizens with difficulties	“This measure alleviated the pressure of workers due to temporary loss of income or income reduction and could be considered as people-friendly”
Securing face mask supply	“Due to the very high demand for masks and limited global supply, the Health Bureau was looking for the supply of masks in different places worldwide… They promised that masks would be supplied immediately once they were available”
Fine-tuning government measures	“To deal with infectious diseases, continuous optimization (of the measures) was needed. Because infectious diseases were unpredictable and uncertain, measures had to be continuously optimized and adjusted. When the epidemic eases, some measures would be changed or withdrawn”
Providing face masks to students and minority	“The measures introduced by the Higher Education Bureau to assist Macao students studying abroad to purchase masks largely alleviated the shortage of protective gear for Macao students studying abroad. The government provided affirmation and support to them in such a time”
Appreciation from different groups of citizens	“More than 95% of the interviewed citizens agreed that the government’s face mask supply program could prevent the spread of the epidemic, reduce the panic of buying masks and play a key role in the fight against the epidemic”

**Table 3:** The most used 10 words in the comments about the face mask scheme (N = word frequency; ec = eigenvector).

Rank	Prodromal Stage	Acute Stage	Chronic Stage
1	pharmacy (N = 26, ec = 0.360)	grateful (N = 192, ec = 0.485)	kids (N = 19, ec = 0.491)
2	sold out (N = 26, ec = 0.047)	cheer up (N = 130, ec = 0.416)	start of the school year (N = 15, ec = 0.313)
3	citizen (N = 13, ec = 0.358)	you (N = 107, ec = 0.486)	go to school (N = 13, ec = 0.218)
4	line up (N = 11, ec = 0.025)	everyone (N = 65, ec = 0.223)	friendly (N = 7, ec = 0.137)
5	convenient (N = 9, ec = 0.077)	hard work (N = 53, ec = 0.283)	problem (N = 6, ec = 0.176)
6	supply (N = 7, ec = 0.115)	frontline staff (N = 48, ec = 0.205)	safe (N = 6, ec = 0.081)
7	flow of people (N = 7, ec = 0.028)	thank you (N = 48, ec = 0.140)	citizen (N = 6, ec = 0.063)
8	quality (N = 6, ec = 0.070)	stay safe (N = 45, ec = 0.169)	tourist (N = 6, ec = 0.049)
9	ID card (N = 5, ec = 0.156)	hope (N = 27, ec = 0.099)	school (N = 5, ec = 0.181)
10	web page (N = 5, ec = 0.084)	salute (N = 24, ec = 0.062)	September (N = 5, ec = 0.163)

**Table 4:** The most used 10 words in the comments about the consumption voucher scheme (N = word frequency; ec = eigenvector).

Rank	Prodromal Stage	Acute Stage	Chronic Stage
1	No words in this stage because the scheme was announced after this stage.	register (N = 5, ec = 0.033)	consumption card (N = 7, ec = 0.332)
2		citizen (N = 4, ec = 0.391)	top up (N = 6, ec = 0.045)
3		original copy (N = 3, ec = 0.234)	first round (N = 5, ec = 0.053)
4		problem (N = 3, ec = 0.028)	register (N = 5, ec = 0.020)
5		application (N = 3, ec = 0.018)	14 days (N = 2, ec = 0.238)
6		Macau Pass (N = 3, ec = 0.017)	balance (N = 2, ec = 0.114)
7		offshore (N = 3, ec = 0.017)	reset to zero (N = 2, ec = 0.114)
8		anti-epidemic (N = 2, ec = 0.382)	merchant (N = 2, ec = 0.107)
9		grateful (N = 2, ec = 0.382)	second round (N = 2, ec = 0.034)

## Data Availability

Data is available upon request.
